# Overinterpretation of high throughput sequencing data in medical genetics: first evidence against *TMPRSS3/GJB2* digenic inheritance of hearing loss

**DOI:** 10.1186/s12967-019-2018-9

**Published:** 2019-08-14

**Authors:** Monika Ołdak, Urszula Lechowicz, Agnieszka Pollak, Dominika Oziębło, Henryk Skarżyński

**Affiliations:** 1 Department of Genetics, World Hearing Center, Institute of Physiology and Pathology of Hearing, M. Mochnackiego 10, 02-042 Warsaw, Poland; 2 Postgraduate School of Molecular Medicine, Medical University of Warsaw, Warsaw, Poland; 3 Oto-Rhino-Laryngology Surgery Clinic, Institute of Physiology and Pathology of Hearing, M. Mochnackiego 10, 02-042 Warsaw, Poland

**Keywords:** Hearing loss, Digenic inheritance, High throughput sequencing, *GJB2*, *TMPRSS3*

## Abstract

**Background:**

Hearing loss (HL) is the most common disability of human senses characterized by a great allelic heterogeneity. *GJB2* and *TMPRSS3* are two well-known HL genes typically underlying its monogenic form. Recently, *TMPRSS3/GJB2* digenic inheritance has been proposed. As results of genetic testing can be easily overinterpreted, we aimed to verify the hypothesis.

**Methods:**

From genetic database of HL patients with at least one *TMPRSS3* pathogenic variants we have selected individuals with additional *GJB2* pathogenic variants. All of the available family members were recruited for the study. Segregation analysis of the respective *TMPRSS3* and *GJB2* pathogenic variants was performed within the families.

**Results:**

The strategy has allowed to identify four individuals who were double heterozygous for known pathogenic *TMPRSS3* and *GJB2* variants. Two individuals from different families had *GJB2* c.35delG and *TMPRSS3* c.208delC and in two other individuals from one family *GJB2* c.35delG together with *TMPRSS3* c.1343T>C variants were found. None of these subjects has ever reported hearing problems and their hearing status was normal.

**Conclusions:**

Our data provide evidence against *TMPRSS3*/*GJB2* digenic inheritance of HL. As high throughput sequencing is increasingly used for genetic testing, particular caution should be taken to provide the patients with accurate genetic counseling.

## Background

Digenic inheritance can be defined as a mechanism, which requires an interaction of two loci for expression of a phenotype. Each of the loci can exert a different level of influence on the phenotype, which means that (i) one locus may represent a primary locus or (ii) both loci may be roughly equal in importance. The first example of digenic inheritance of human diseases is retinitis pigmentosa attributed to recessive variants in the *PRPH2* and *ROM1* genes [1]. Their causative role was provided based on convincing data from different family studies and confirmed interaction between the two gene products.

The growing number of human disorders with a digenic pattern of inheritance is now being collected in digenic diseases database (DIDA), a comprehensive repository with records on genes and genetic variants involved in digenic diseases (http://dida.ibsquare.be/). Hereditary hearing loss (HL) is a genetically heterogenous condition with over 100 genes involved in its development, which makes HL a good candidate for digenic inheritance. Congenital hereditary hearing impairment is a common disorder which affects approx. 1:2000 neonates. The major genetic cause of HL are biallelic *GJB2/GJB6* (DFNB1 locus) pathogenic variants [2]. Another important contributor to autosomal recessive non-syndromic HL is the *TMPRSS3* gene [3].

In the study we have aimed to verify the hypothesis of putative *TMPRSS3*/*GJB2* digenic inheritance of HL. The idea was precipitated by recent publications suggesting the novel gene combination involved in HL development and reinforced by inquires from health care professionals and patients on how to interpret the co-occurrence of heterozygous recessive variants in both genes. According to previous reports up to one-third of “solved cases” may be attributable to overinterpretation due to incorrect assessment of variant pathogenicity or poor-evidenced digenic or oligogenic inheritance [4–6].

## Methods

### Patients

The study was approved by the local ethics committee (IFPS:/KB/03/2012). From patients tested for *TMPRSS3* pathogenic variants (n = 2277) we have first selected individuals with at least one* TMPRSS3* pathogenic variant (n = 42) and next patients with additional pathogenic variants in the* GJB2* gene (n = 4). The probands and their family members (n = 18) gave written informed consent for participation in the study. Detailed medical history was evaluated. Hearing status was determined by self-assessment or pure tone-audiometry with hearing thresholds measured at selected frequencies 125, 250, 500, 1000, 2000, 4000 and 6000 Hz using the AC40 clinical audiometer (Interacoustics, Middelfart, Denmark). Hearing thresholds up to 20 dB are considered to be normal hearing. DNA was isolated from blood samples or buccal swabs using a standard protocol. Presence of the respective* TMPRSS3* (NM_024022.2, NP_076927.1) and* GJB2* (NM_004004.5, NP_003995.2) pathogenic variants was verified by Sanger sequencing [3] and family segregation study was performed.

### Databases

DIDA was used to obtain information on genetic variants involved in digenic HL (http://dida.ibsquare.be/; accessed 10/2018). Interaction networks were generated using STRING v.10.5 (https://string-db.org/; accessed 12/2018), a database of known and predicted protein–protein interactions. Expression pattern of *GJB2* and* TMPRSS3* in the inner ear was collected from previous studies (Table 1).

**Table 1:** Expression pattern of *GJB2* and *TMPRSS3* genes

Inner ear structure	*GJB2* expression		*TMPRSS3* expression	
	Rodents [22]	Human [9]	Rodents [23, 24]	Human [10]
Inner hair cells	−	−	+	+
Outer hair cells	−	−	±	+
Interdental cells	+	N/A	+	N/A
Spiral limbus	+	N/A	−	N/A
Inner sulcus cells	+	N/A	+	N/A
Inner pillar cells	+	−	−	+
Outer pillar cells	+	+	−	+
Deiter’s cells	+	+	+	+
Hensen cells	+	+	+	N/A
Claudius cells	+	+	+	N/A
External sulcus cells	+	N/A	+	N/A
Stria vascularis	+	+	+	±
Spiral ligament	+	+	−	N/A
Spiral ganglion	−	+	+	±

−, no expression detected; +, detected expression; ±, inconsistent expression data; N/A, no data available

## Results

**Fig. 1:**
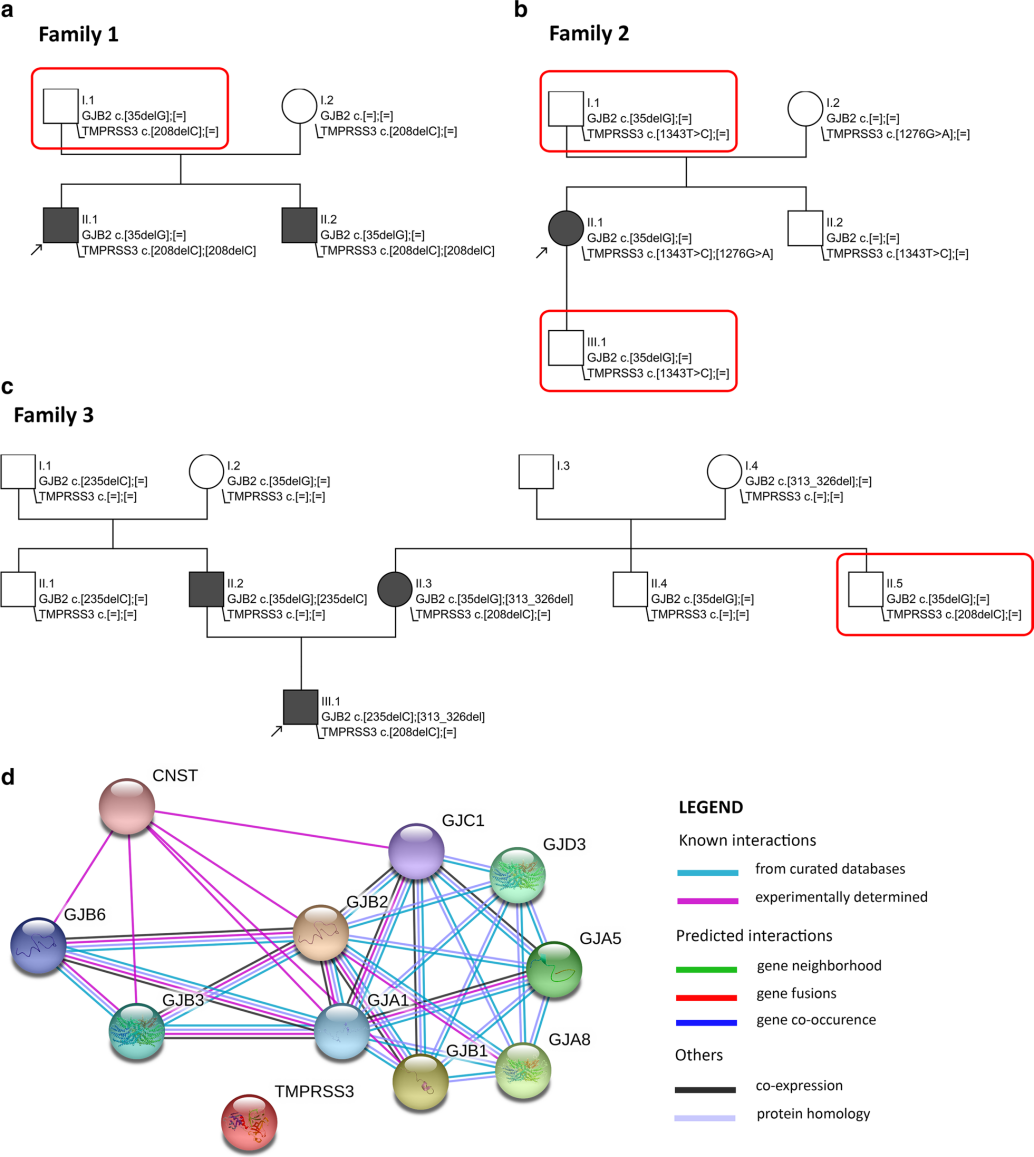
Coexistence of *GJB2* and *TMPRSS3* pathogenic variants in normal hearing individuals supports lack of interactions between the two genes. **a**–**c** Pedigrees of the analyzed families. Probands are marked with an arrow. Black symbols indicate individuals affected with HL and open symbols indicate unaffected individuals. Red rectangles mark normal hearing individuals being double heterozygous for *GJB2* and *TMPRSS3* pathogenic variants. **d** Map of *GJB2* and *TMPRSS3* interactions according to STRING v.10.5 database. Round symbols indicate different genes. Interactions between genes are represented by color lines described in the legend

In Family 1 prelingual profound bilateral sensorineural HL was diagnosed in the proband and his brother. They received cochlear implants (CI) at the age of 4 y and 2 y, respectively. Genetic testing revealed that HL in the family was a consequence of homozygous pathogenic c.208delC (p.His70Thrfs*19) variant in the* TMPRSS3* gene. Carrier status of the* TMPRSS3* variant was confirmed in both parents. In addition to that both HL siblings and their normal hearing father (hearing status confirmed by pure tone audiometry) were carriers of a pathogenic heterozygous c.35delG (p.Gly12Valfs*2) variant in the *GJB2* gene. Neither paternal grandparents nor his siblings were available for genetic testing but none of them have complained of hearing impairment (Fig. 1a).

The proband in Family 2 had progressive bilateral sensorineural HL affecting mainly high frequencies from the age of about 20 years. She received hearing aids at the age of 30 y and CI of the right ear was performed at the age of 40 y. The patient was compound heterozygous for two pathogenic variants, i.e. c.1276G>A (p.Ala426Thr) and c.1343T>C (p.Met448Thr) in the* TMPRSS3* gene that were inherited from her normal hearing parents. The* TMPRSS3* c.1343T>C variant was detected also in the proband’s father, brother and her 23-year old normal hearing son. Except for the* TMPRSS3* variants the proband, her father and her son were also heterozygous for the pathogenic* GJB2* c.35delG variant (Fig. 1b).

Family 3 was characterized by prelingual profound bilateral sensorineural HL diagnosed in the proband and both of his parents. The proband received CI at the end of the first year of life. He is compound heterozygous for *GJB2* c.235delC (p.Leu79Cysfs*3) and c.313_326del (p.Lys105Glyfs*5) pathogenic variants. HL in the proband’s father resulted from two *GJB2* pathogenic variants c.35delG and c.235delC in a trans configuration. Proband’s mother was compound heterozygous for *GJB2* c.35delG and c.313_326del pathogenic variants. In addition to the *GJB2* variants, the proband and his mother were also carriers of a *TMPRSS3* c.208delC pathogenic variant. A combination of one *TMPRSS3* (c.208delC) and one *GJB2* (c.35delG) variant was detected in the proband’s maternal uncle, who does not have hearing impairment. Both, proband’s mother and her brother, have most probably inherited the combination of *GJB2* and *TMPRSS3* variants from their normal hearing father, who was not available for genetic testing (Fig. 1c).

In DIDA there are two records on possible *TMPRSS3*/*GJB2* digenic inheritance of HL. The first one refers to a combination of *GJB2* c.35delG together with *TMPRSS3* c.208delC for which only familial evidence (based on one family) has been provided by Battelino et al. [7]. The second combination is *GJB2* c.487A>G (p.Met163Val) and *TMPRSS3* c.1276G>A (p.Ala426Thr) that is supposed to be evidenced by familial and functional data [8].

Detailed expression pattern of *GJB2* and *TMPRSS3* has been presented in Table 1. It overlaps partially and is restricted mainly to supporting cells. *GJB2* encodes connexin 26, a gap junction protein, which forms cell-to-cell channels permeable in the inner ear to K^+^ ions and other small molecules [9]. *TMPRSS3* is a transmembrane protein with protease activity. Recent studies indicate that it is involved in actin-related hair cell mechanics and support of cell motility and integrity [10]. Currently, there is no evidence of direct relationships between the two genes. In STRING neither known (from curated databases or experimentally determined) nor predicted direct interactions between *TMPRSS3* and *GJB2* genes and proteins was reported (Fig. 1d).

## Discussion

Based on family studies we have identified as many as four individuals from three different HL families who were double heterozygous for pathogenic variants in the *GJB2* and *TMPRSS3* genes (Fig. 1a–c). Interestingly, none of the subjects was diagnosed with HL. Our data provide strong evidence against *TMPRSS3*/*GJB2* digenic inheritance of HL that has been proposed by two recent reports [7, 8], which in our opinion are far from being convincing. Both studies rely exclusively on data from only two families with either one [7] or two [8] affected individuals carrying heterozygous *GJB2* and *TMPRSS3* variants.

In the study by Battelino et al. one patient has been proposed to have HL due to digenic inheritance of *GJB2* c.35delG and *TMPRSS3* c.208delC. However, a second variant c.579dupA in *TMPRSS3* was also detected but it was erroneously interpreted as occurring in the non-coding sequence and being non-pathogenic. As noticed by our group [11] congenital profound HL in the patient is not a consequence of *TMPRSS3*/*GJB2* digenic inheritance but results from compound heterozygous c.208delC (p.His70Thrfs*19) and c.579dupA (p.Cys194Metfs*17) *TMPRSS3* pathogenic variants.

The second paper on the presumed *TMPRSS3*/*GJB2* digenic inheritance of HL presents two siblings with moderate-to-severe HL at mid and high frequencies. Sequencing of 71 known HL genes revealed a heterozygous *GJB2* c.487A>G (p.Met163Val) variant and a heterozygous *TMPRSS3* c.1276G>A (p.Ala426Thr) variant. Hearing status of the patients' parents was normal and each of them carried one of the detected variants [8]. While the *TMPRSS3* variant was found as causative of postlingual progressive HL with recessive mode of inheritance [3] and its pathogenic potential was confirmed by in vitro studies [12], the pathogenic role of the *GJB2* p.Met163Val variant is still intriguing. Up to now it has been detected in approximately 18 HL patients. In the majority of them p.Met163Val was identified in a simple heterozygous state and its pathogenicity was described as unknown. In vitro studies have shown that p.Met163Val affects the formation of homotypic connexin 26 junctional channels [13]. According to ACMG/AMP standards and guidelines for the interpretation of sequence variants, p.Met163Val should be classified as likely pathogenic [14].

There are at least three different possible explanations for the genetic cause of HL (other than *TMPRSS3*/*GJB2* digenic inheritance) in the family studied by Leone et al. [8]. The first option is a dominant character of the identified *GJB2* p.Met163Val variant with a possible incomplete penetrance in the patients’ mother. Another alternative is the omission of the second pathogenic DFNB1 variant due to e.g. poor quality sequencing, incomplete coverage or presence of undetected copy number variants. The third option is the involvement of genes other than *GJB2* or *TMPRSS3*. Testing of a limited number of HL genes does not exclude the possibility that the genetic cause of HL is located in the untargeted regions of these genes such as alternative exons, introns or regulatory regions as well as in other known (currently more than 100 HL genes have been discovered) or still unknown HL genes or regulatory RNAs.

Among the relationships determining a presumed digenic inheritance between a pair of genes are their direct or indirect interactions, involvement in a common pathway, co-expression or similar function [15]. This should be taken into account if a digenic combination segregates with phenotype in a family. Based on what is currently known about the expression pattern, biological function or interaction between *GJB2* and *TMPRSS3*, not only in the inner ear but also in other organs, it is difficult to find a common denominator for *GJB2* and *TMPRSS3* that could justify a phenomenon of their digenic inheritance. To experimentally test the hypothesis of *TMPRSS3*/*GJB2* digenic inheritance, introduction of animal models and performing extensive cell biology experiments would be relevant [16–19]. In our opinion detection of double heterozygous *TMPRSS3*/*GJB2* variants in HL patients is a purely coincidental finding. The carrier frequency of *GJB2* variants in the general population has been estimated at approximately 3% [2] while *TMPRSS3* at 0.4% (Lechowicz U. unpublished data). Considering the frequencies, one should expect a higher prevalence of HL due to digenic *TMPRSS3*/*GJB2* inheritance than due to *TMPRSS3* pathogenic variants alone. Since the publication by Leone et al. [8] there are no other reports confirming the presumed new phenomenon of *TMPRSS3*/*GJB2* digenic inheritance. *GJB2* is the most common cause of hereditary HL and one should keep in mind that it can be frequently find in tested individuals. Moreover, there is a significant enrichment of simple heterozygous *GJB2* pathogenic variants in HL patients (~ 5% vs 2-3% in the general population) [20]. This observation may be a consequence of a higher complexity of the DFNB1 locus than currently accepted or related to an increased number of pathogenic variants in HL genes found in HL patients, referred to as mutational load [21].

## Conclusion

High throughput sequencing data in HL patients may suggest a more complex oligogenic inheritance of HL but our results provide evidence that simple co-occurrence of heterozygous *GJB2* and *TMPRSS3* recessive variants is not related to the development of disabling HL. The number of detected, probably pathogenic variants increases with the use of advanced sequencing techniques, which may lead to overinterpretation of genotyping results. Particular caution and use of specific guidelines is required by data analysis and selection of causative variants in order to provide accurate diagnosis and counseling for the patients and reliable data for the medical genetic community [5, 14].

## Data Availability

Please contact author for data requests.

## Abbreviations

HL: hearing loss
ACMG/AMP: the American College of Medical Genetics and Genomics and the Association for Molecular Pathology
DIDA: digenic diseases database
STRING: Search Tool for the Retrieval of Interacting Genes/Proteins
